# Progress against inequalities in mortality: register-based study of 15 European countries between 1990 and 2015

**DOI:** 10.1007/s10654-019-00580-9

**Published:** 2019-11-15

**Authors:** Johan P. Mackenbach, José Rubio Valverde, Matthias Bopp, Henrik Brønnum-Hansen, Giuseppe Costa, Patrick Deboosere, Ramune Kalediene, Katalin Kovács, Mall Leinsalu, Pekka Martikainen, Gwenn Menvielle, Maica Rodriguez-Sanz, Wilma J. Nusselder

**Affiliations:** 1 Department of Public Health, Erasmus MC, P.O. Box 2040, 3000 CA Rotterdam, The Netherlands; 2 Epidemiology, Biostatistics and Prevention Institute, University of Zürich, Zurich, Switzerland; 3 Institute of Public Health, Copenhagen University, Copenhagen, Denmark; 4 Department of Clinical Medicine and Biology, University of Turin, Turin, Italy; 5 Department of Sociology, Vrije Universiteit Brussel, Brussels, Belgium; 6 Lithuanian University of Health Sciences, Kaunas, Lithuania; 7 Demographic Research Institute, Budapest, Hungary; 8 Stockholm Centre for Health and Social Change, Södertörn University, Stockholm, Sweden; 9 Department of Epidemiology and Biostatistics, National Institute for Health Development, Tallinn, Estonia; 10 Department of Sociology, University of Helsinki, Helsinki, Finland; 11 INSERM, Sorbonne Universités, Institut Pierre Louis d’Epidémiologie et de Santé Publique (IPLESP UMRS 1136), Paris, France; 12 Agència de Salut Pública de Barcelona, Barcelona, Spain; 13 CIBER de Epidemiología y Salud Pública (CIBERESP), Madrid, Spain

**Keywords:** Mortality, Social inequality, Trends, Europe

## Abstract

Socioeconomic inequalities in mortality are a challenge for public health around the world, but appear to be resistant to policy-making. We aimed to identify European countries which have been more successful than others in narrowing inequalities in mortality, and the factors associated with narrowing inequalities. We collected and harmonised mortality data by educational level in 15 European countries over the last 25 years, and quantified changes in inequalities in mortality using a range of measures capturing different perspectives on inequality (e.g., ‘relative’ and ‘absolute’ inequalities, inequalities in ‘attainment’ and ‘shortfall’). We determined which causes of death contributed to narrowing of inequalities, and conducted country- and period-fixed effects analyses to assess which country-level factors were associated with narrowing of inequalities in mortality. Mortality among the low educated has declined rapidly in all European countries, and a narrowing of absolute, but not relative inequalities was seen in many countries. Best performers were Austria, Italy (Turin) and Switzerland among men, and Spain (Barcelona), England and Wales, and Austria among women. Ischemic heart disease, smoking-related causes (men) and amenable causes often contributed to narrowing inequalities. Trends in income inequality, level of democracy and smoking were associated with widening inequalities, but rising health care expenditure was associated with narrowing inequalities. Trends in inequalities in mortality have not been as unfavourable as often claimed. Our results suggest that health care expansion has counteracted the inequalities widening effect of other influences.

**Electronic supplementary material:**

The online version of this article (10.1007/s10654-019-00580-9) contains supplementary material, which is available to authorized users.

## Introduction

Inequalities in mortality by socioeconomic position are among the most consistently reproduced findings in public health research: rates of mortality are higher among those with a lower education, occupational class or income in all countries that have taken the trouble to collect the necessary data [1–3]. The ubiquity and persistence of health inequalities, despite the rise of the welfare state and some countries’ explicit attempts to narrow them, even give the impression that health inequalities are resistant to policy-making [4].

However, with data on more countries and longer periods of time becoming available, it is increasingly becoming clear that these inequalities are more variable and dynamic than is often appreciated. Within Europe, inequalities in mortality are smaller in the Mediterranean region, and larger in the former East bloc, with Northern Europe also having surprisingly large inequalities in mortality, particularly among women [1]. In many countries, relative inequalities in mortality (as indicated by, e.g., the rate ratio of mortality among lower as compared to higher socioeconomic groups) have risen strongly, whereas absolute inequalities (as indicated by, e.g., the difference between the mortality rates of lower and higher socioeconomic groups) have followed a more variable course [5].

These variations between countries and over time are important because they show that the magnitude of inequalities in mortality is far from fixed, and that there is, in principle, large scope for reducing health inequalities. In this paper, we aim to identify the European countries which have been more successful than others in containing or even reducing inequalities in mortality, and we exploit variations between countries to identify the factors associated with narrowing and widening of inequalities in mortality.

## Methods

### Data

We collected and harmonized register-based mortality data from 15 European countries covering all parts of the subcontinent. Most data cover complete national populations with the exceptions of England and Wales and France (1% representative samples) and Spain and Italy (Barcelona and Turin only). Most data stem from a longitudinal mortality follow-up after a census. An overview of the mortality data sources and some key characteristics of the data are given in Web appendix Table A1. Web appendix Table A2 gives details on the causes of death used in the analyses.

Socioeconomic position was indicated by highest level of completed education, in three categories: ‘low’, ‘mid’, and ‘high’ (corresponding to ISCED 1997 categories 0–2, 3–4 and 5–6, respectively). We focused on educational inequalities (instead of, e.g., occupational inequalities) because comparable data were available for a wider range of countries, and because education is normally completed in early adulthood, which avoids most problems of reverse causation [6]. The analyses were restricted to ages 35–79 years because at advanced ages the proportion of high educated becomes very small, and education becomes a less sensitive indicator of socioeconomic position.

For our analysis of possible determinants of trends in mortality inequalities we extracted four national-level variables from harmonised international databanks, each of which has been shown to be associated with mortality in cross-national analyses in Europe: national income (natural logarithm of Gross Domestic Product (GDP) per capita, measured in purchasing power parity adjusted US$; source: World Health Organization Health for All Database; higher income is associated with lower mortality [7]), income inequality (Gini index of net equivalised household income; source: Standardizing the World Income Inequality Database; higher income inequality is associated with higher mortality [8]), level of democracy (combined Freedom House—Polity2 democracy score; source: Quality of Government Dataset; transition to democracy is associated with a temporary rise in mortality [9]), and health care expenditure (current expenditure on health as a percentage of GDP; source: World Health Organization Health for All Database; higher health care expenditure is associated with lower mortality [10]).

We also collected and harmonized data on two determinants of mortality that were available by level of education: smoking (current cigarette smoking; source: national health interview surveys; higher prevalence of smoking is associated with higher mortality [11]) and material deprivation (inability to pay for at least three out of nine items deemed necessary for a normal life; source: EU Statistics on Income and Living Conditions survey; higher prevalence of material deprivation is associated with higher mortality [11]).

### Analysis

Mortality rates by educational level were directly age-standardized using the European Standard Population [12] and five-year age-groups. We also calculated partial life expectancies between the ages of 35 and 80 (years of life lived, as a measure of ‘attainment’) and differences between partial life expectancies and the theoretical maximum of 45 years (years of life lost, as a measure of ‘shortfall’) [13].

With these outcomes we calculated a total of eight measures of inequalities in mortality, capturing different perspectives on inequality: Rate Differences and Rate Ratios of mortality rates, Slope Indices of Inequality and Relative Indices of Inequality (which, in contrast to simple Rate Ratios and Rate Differences, make an adjustment for shifts in the educational distribution [14, 15]), Differences and Ratios of years of life lived, and Differences and Ratios of years of life lost. We refrained from adding even more measures, such as the recently developed Slope Index of Inequality for years of life lost [16]. Please note that absolute differences of years of life lived and years of life lost are numerically identical. We used a wide range of measures of inequalities in mortality to counter recent criticism against reliance on a more restricted set of measures that is biased towards finding widening inequalities [13].

In order to analyse the relationship between determinants and inequalities in mortality we conducted panel (or pooled cross-sectional time series) analyses in which mortality was modelled as a function of national-level determinants, using country- and period-fixed effects, in the following way:

1 $${\text{M}}_{\text{ect}} = \upalpha +\upbeta_{1} *{\text{C}} +\upbeta_{2} *{\text{P}} + \upbeta_{3} *{\text{D}}_{\text{ct}} + \upbeta_{4} *{\text{E}} + \upbeta_{5} *{\text{D}}_{\text{ct}} *{\text{E}} + {\text{u}}_{{0{\text{ct}}}} + \upvarepsilon_{\text{ect}}$$

in which M = age-standardized mortality rate, C = a set of dummy variables representing country fixed effects, P = a set of dummy variables representing period fixed effects, D = determinant, E = level of education, α = intercept, β_1_ and β_2_ = parameters indicating differences in levels of M between countries and periods, β_3_ = parameter indicating main effect of determinant on M, β_4_ = parameter indicating main effect of education on M, β_5_ = parameter indicating effect of interaction between determinant and education on M, u = country- and period-specific random residual, ε = education-specific random residual, e = subscript denoting level of education, c = subscript denoting country, t = subscript denoting calendar-year.

For the determinants that were available by level of education, i.e., smoking and material deprivation, we conducted similar analyses, in the following way:

2 $${\text{M}}_{\text{ect}} = \upalpha + \upbeta_{1} *{\text{C}} + \upbeta_{2} *{\text{P}} + \upbeta_{3} *{\text{D}}_{\text{ect}} + \upbeta_{4} *{\text{E}} + {\kern 1pt} {\text{u}}_{{0{\text{ct}}}} + \upvarepsilon_{\text{ect}}$$

All regression analyses were done with the SPSS 24 routine for linear mixed models. This allowed us to apply a multilevel framework and thus to take into account dependency of data within countries and time-periods. We used an autoregressive model of order 1 to take into account the year-on-year serial autocorrelation in the mortality rates.

### Role of the funding source

The study sponsor had no role in study design, collection, analysis, and interpretation of data, writing of the report, or in the decision to submit the paper for publication.

## Results

**Fig. 1:**
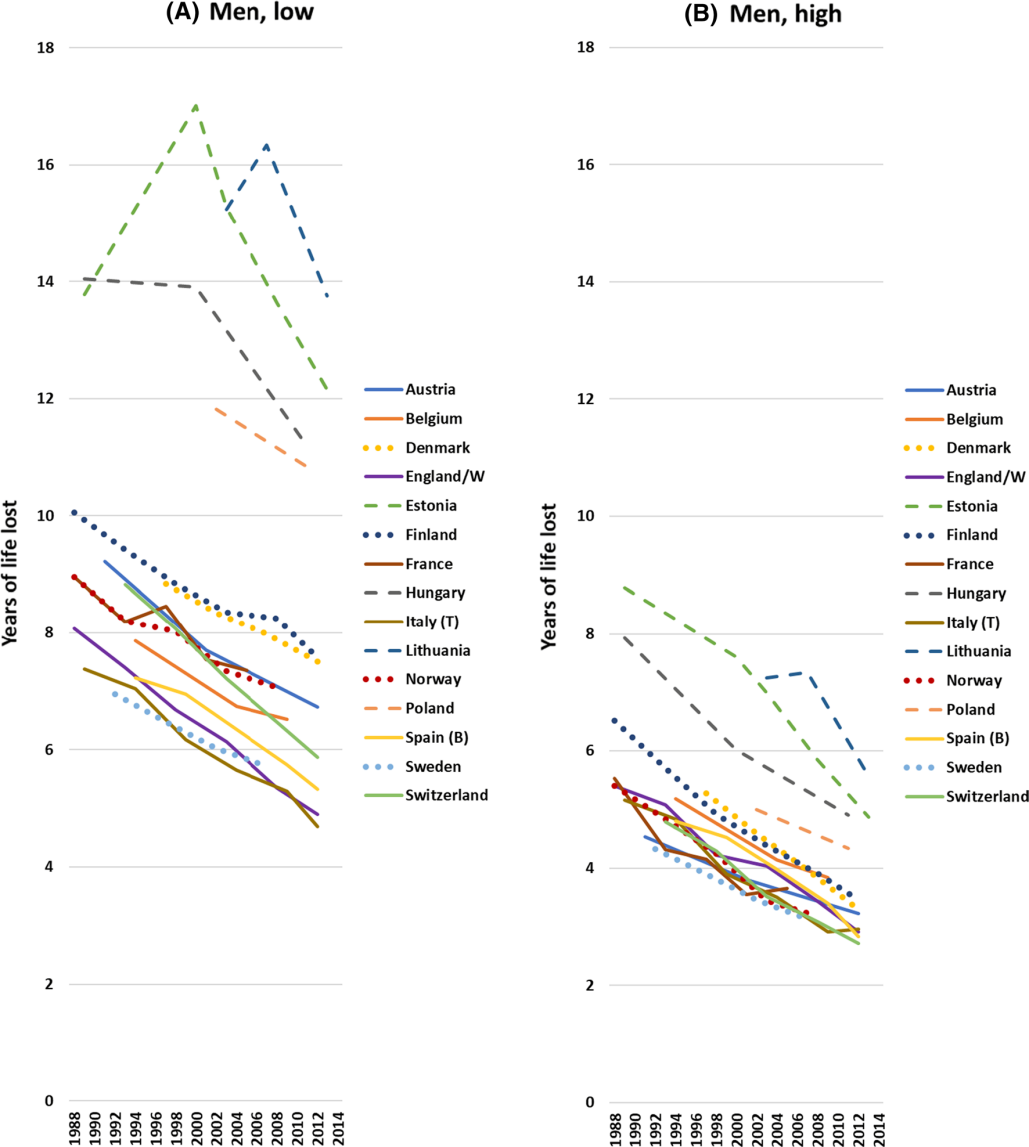

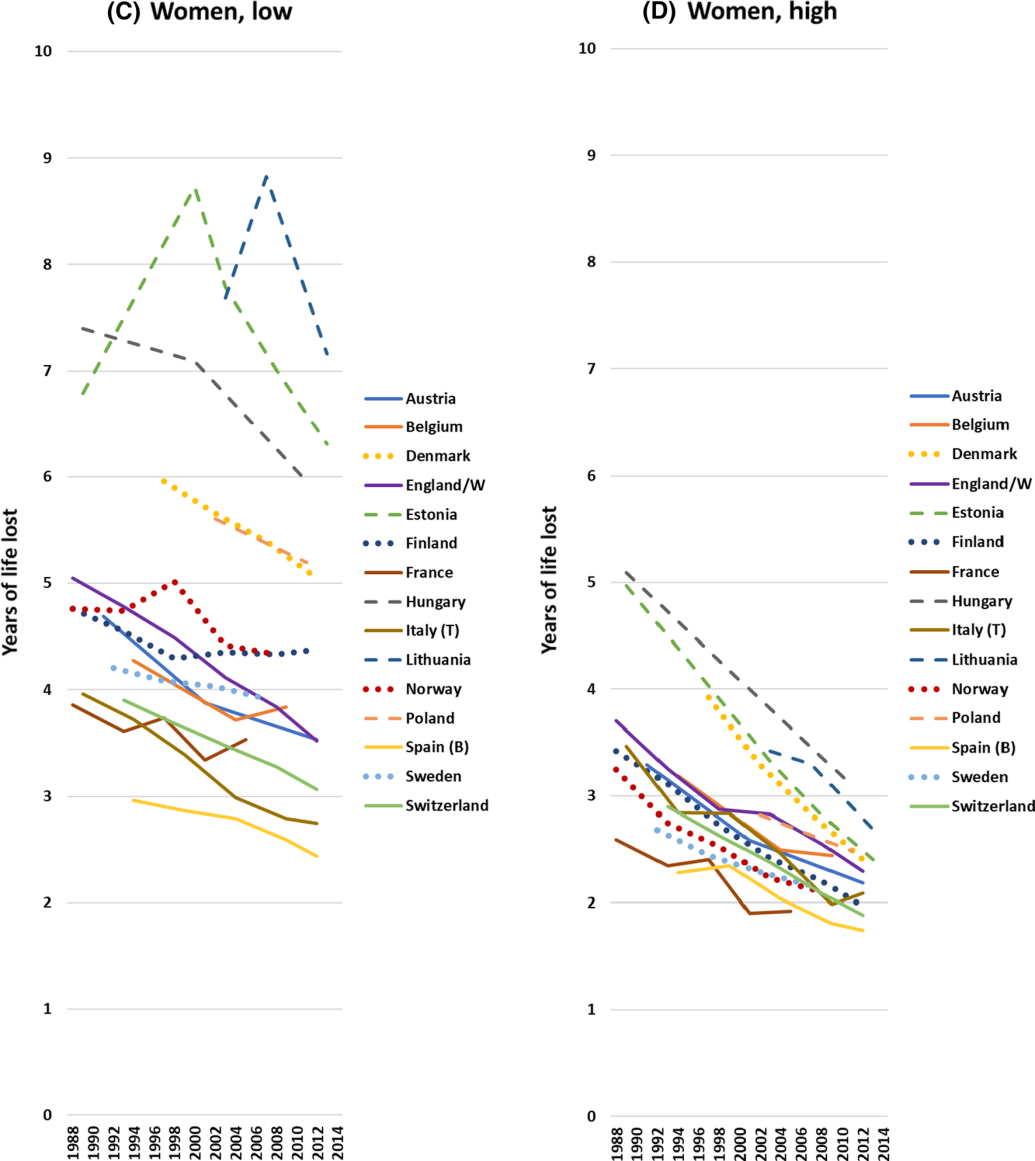
Trends in years of life lost between the ages of 35 and 80 years, by country, gender and level of education. Notes Years of life lost calculated as partial life expectancy (between the ages of 35 and 80 years) minus 45 years. Dashed lines: Eastern European countries; dotted lines: Northern European countries. Scale for men and women different

Trends in mortality have generally been favourable, both for the low and the high educated, and regardless of whether we study mortality rates, years of life lived or years of life lost. We illustrate this in Fig. 1 with data on years of life lost between the ages of 35 and 80. In Northern, Western and Southern Europe, these shortfalls in life expectancy have steadily declined over this time-period, both among the low and high educated, but in Eastern Europe recent improvements represent a reversal as compared to unfavourable trends up to around the year 2000 (full details in Web appendix Table A3).

**Fig. 2:**
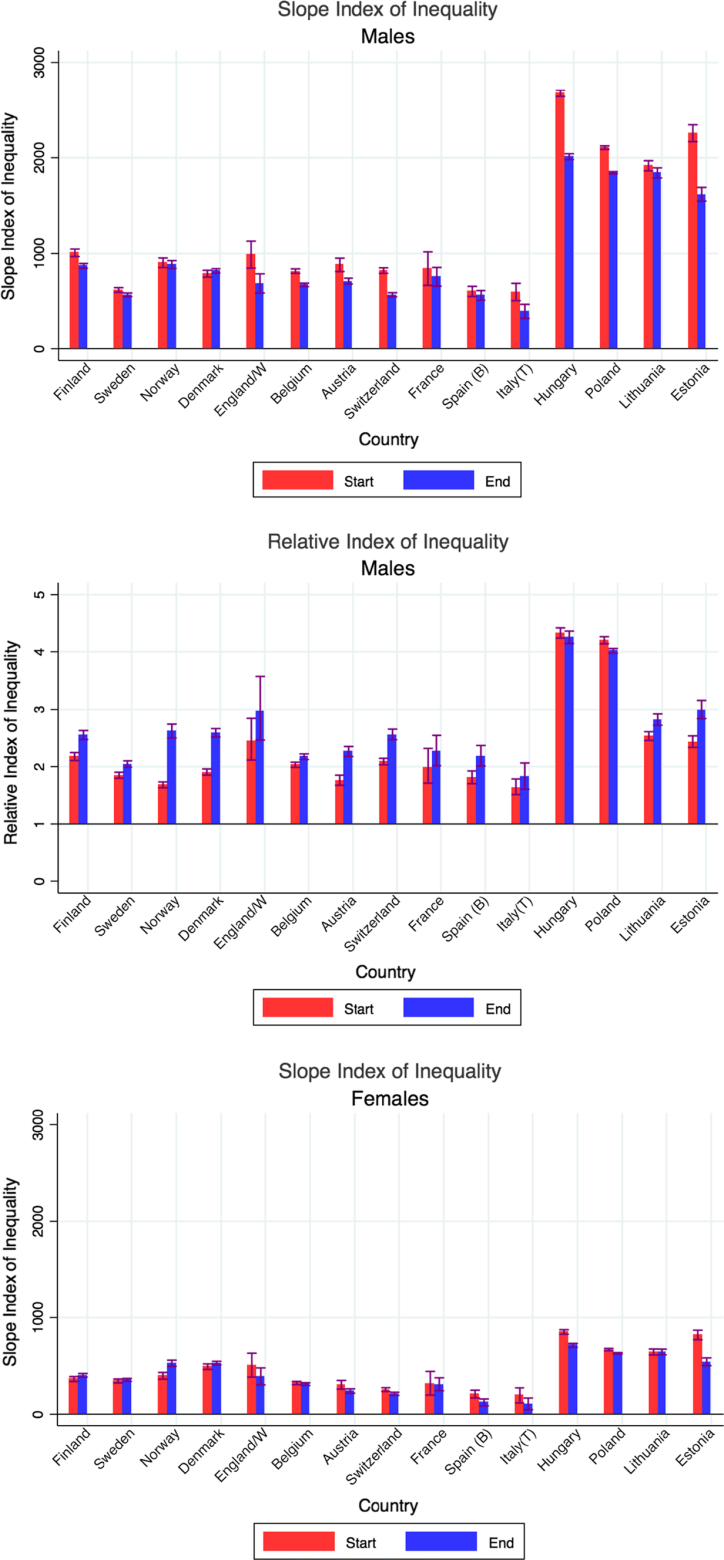

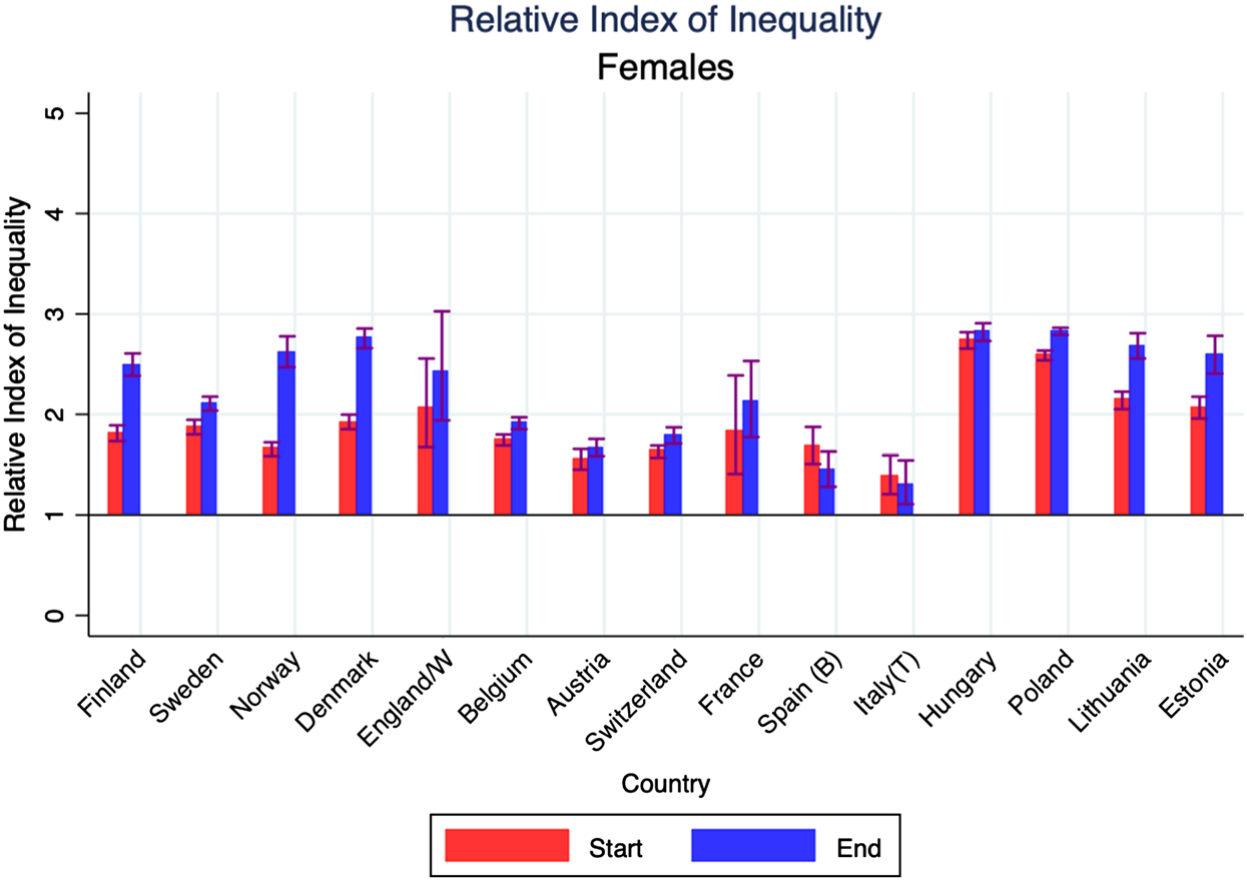
Changes in Slope Index of Inequality and in Relative Index of Inequality for mortality, by country and gender. Notes: Start = around 1990; end = around 2012. *Start = around 2000; end = around 2012. For exact timing of observation periods, see Table 1 and Web appendix Table A1

Mortality has always been higher, and life expectancy lower, among the low than the high educated, but due to differences in the rate of improvement between low and high educated, gaps have sometimes narrowed, sometimes widened over time. We illustrate this in Fig. 2 with Slope and Relative Indices of Mortality. Among men, the Slope Index of Inequality (SII), which measures absolute differences in mortality rates between education groups taking into account their relative sizes, has mostly gone down, whereas the Relative Index of Inequality (RII) has mostly gone up. Among women, the trends are more variable, but here too the trends in absolute inequalities (SII) are much more favourable than trends in relative inequalities (RII).

Whether a narrowing or widening has occurred is strongly dependent on the measure used. Table 1 presents the changes in all eight measures of inequality, and while it confirms that trends in absolute inequalities have usually been more favourable than trends in relative inequalities, it also shows that the reverse is the case for years of life lived, for which relative differences sometimes narrowed even though absolute differences widened.

**Table 1:**
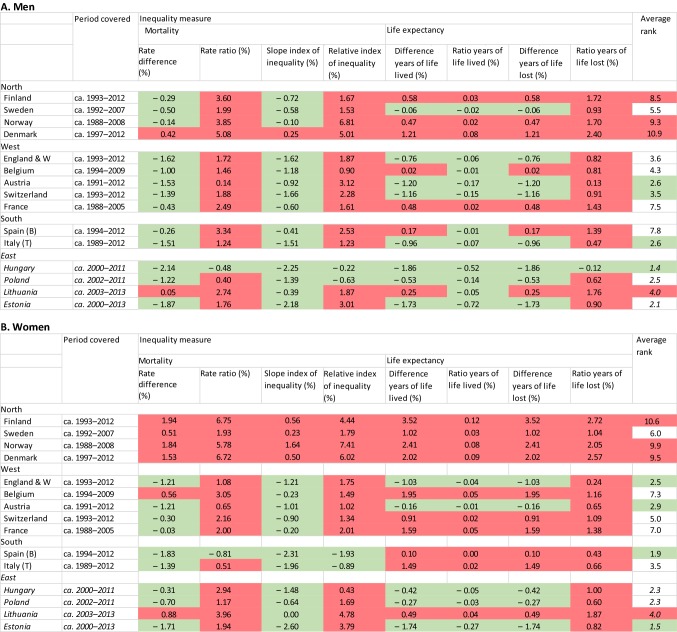
Annual changes in eight quantitative measures for the magnitude of inequalities in mortality

For each inequality measure, this table presents per cent per annum changes calculated between beginning and end of the observation period. For Eastern Europe (in italics) changes have been calculated over a more recent period, with declining mortality, only. In green: narrowing of inequalities in mortality; in red, widening of inequalities in mortality. Each inequality measure was also used to calculate a country’s rank (with 1 representing the country with most narrowing or least widening of inequalities), and then to calculate a country’s average rank over all eight inequality measures (with lower values indicating more narrowing or less widening; see Web appendix Table A4). In green: best performing countries; in red: worst performing countries

However, ranking of countries in terms of more or less narrowing of inequalities in mortality is hardly dependent on the measure used. This ranking is presented in Web appendix Table A4 and the last column of Table 1. Because mortality in Eastern Europe started to decline only recently, these countries have been ranked separately.

Combining all eight measures of inequalities in mortality, Austria, Italy (Turin) and Switzerland have had the most favourable trends among men, whereas Spain (Barcelona), England and Wales, and Austria have had the most favourable trends among women. On the other end of the scale, Finland, Norway, and Denmark have seen the least narrowing of inequalities in mortality. In Eastern Europe, Hungary (men) and Estonia (women) performed best, and Lithuania performed worst.

**Fig. 3:**
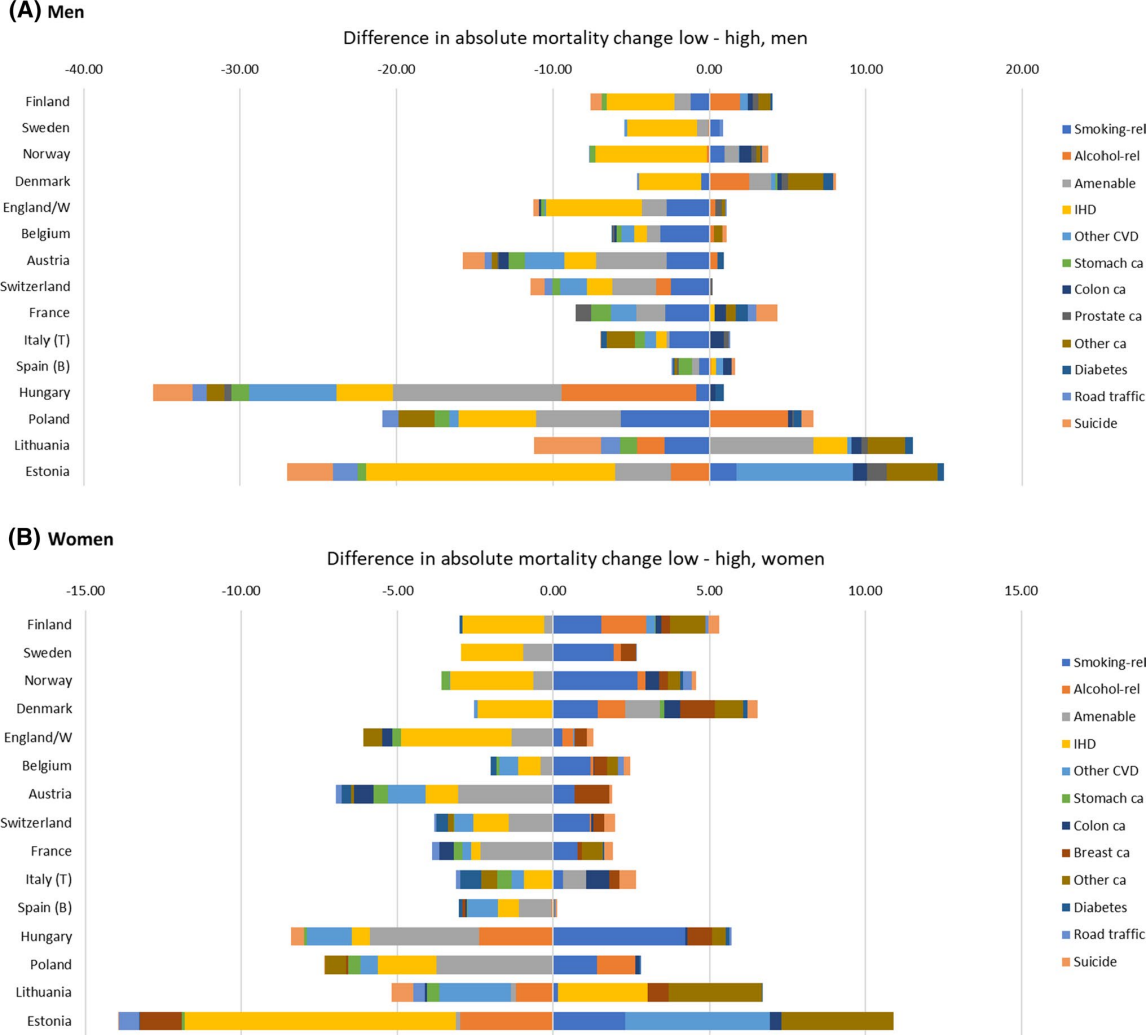
Contribution of 13 causes of death to changes in absolute inequalities in mortality between low and high educated. **a** Men, **b** Women. Notes: Difference between low and high expressed in annual mortality change, expressed as deaths per 100,000 person-years. For Eastern Europe changes have been calculated over the most recent observation period, with declining mortality. Excluding all other causes of death

What drives differences between low and high educated in the pace of mortality decline? As a first step, we have identified the causes of death for which mortality decline has been faster or slower among the low than among the high educated (Fig. 3). The 13 causes of death included in this analysis present a highly variable picture. For example, absolute declines in mortality from ischemic heart disease and amenable causes were mostly faster among the low than among the high educated (shown as negative values in Fig. 3), whereas declines in mortality from breast cancer and other cancers have mostly been faster among the high than among the low educated (shown as positive values in Fig. 3).

Differences between countries in the extent of narrowing of inequalities in total mortality are partly dependent on whether or not there are causes of death for which declines in mortality have been larger among the high than the low educated, instead of vice versa. For example, Austria’s very good performance is due to almost uniformly larger declines in mortality among the low educated. At the other end of the spectrum, Denmark’s bad performance is due to the fact that for many causes of death declines in mortality have been larger among the high than the low educated. The excellent performance of Hungary (men) is based on massive reductions among the low as compared to the high educated in mortality from, among others, amenable causes and alcohol-related causes.

As we have seen, trends in inequalities in mortality are less favourable among women than among men. Figure 3 shows that this is partly due to the fact that declines in mortality from smoking-related causes are mostly larger among the high than the low educated among women, whereas the reverse is true for men. The worse performance of the Nordic countries, however, is not only due to more unfavourable trends among the low educated in smoking-related mortality, but also to more unfavourable trends for other causes, including alcohol-related mortality and (in the case of Denmark) amenable causes.

As a second step, Table 2 presents the results of regression analyses in which we analysed which determinants of mortality were associated with a widening or narrowing of absolute inequalities in mortality. Trends in national income, income inequality, levels of democracy and health care expenditure have, on average, all been upwards, but their associations with mortality and mortality inequalities have been different. Rising national income was associated with declining mortality, but in absolute terms the relationship was not statistically significantly different between low and high educated, implying that absolute inequalities in mortality are not affected by rising national income. Rising income inequality was associated with rising mortality, particularly among men, and this relationship was stronger for the low educated, suggesting that rising income inequality may have contributed to widening inequalities in mortality. Rising levels of democracy were associated with rising mortality among the low educated, again suggesting a mortality widening effect. By contrast, rising health care expenditure was associated with stronger mortality decline among the low educated, implying that this may have contributed to a narrowing of inequalities in mortality.

**Table 2:** Results of regression analyses

	Effect of time on determinant in whole population		Main effect of determinant on mortality		Interaction effect between determinant and low education on mortality		Effect of trend in determinant on mortality inequalites	
	Men	Women	Men	Women	Men	Women	Men	Women
National income	0.043***	0.043***	− 338***	− 78 (n.s.)	− 69.4 (n.s.)	15.0 (n.s.)	None	None
Income inequality	0.158***	0.158***	16.7***	0.6 (n.s.)	37.2***	18.5***	Widening	Widening
Level of democracy	0.105***	0.105***	1.1 (n.s.)	− 23.7***	90.8***	74.3***	Widening	Widening
Health expenditure	0.124***	0.124***	− 29.2 (n.s.)	− 8.9 (n.s.)	− 168***	− 53***	Narrowing	Narrowing
Control variables	c, e	c, e	c, p, e, g	c, p, e, g	c, p, e, g	c, p, e, g		

	Effect of time on determinant in whole population		Interaction effect between time and low education determinant		Effect of determinant on mortality		Effect of trend in determinant on mortality inequalities	
	Men	Women	Men	Women	Men	Women	Men	Women
Smoking	− 0.004***	0.000 (n.s.)	0.005***	0.007***	1274***	361***	Widening	Widening
Material deprivation	− 0.002 (n.s.)	− 0.001 (n.s.)	0.001 (n.s.)	0.001 (n.s.)	1134***	265***	None	None
Control variables	c, e	c, e	c, e, y	c, e, y	c, e, y	c, e, y		

Control variables: c = country, p = period, e = education, g = GDP, y = year. In bold: statistically significant (*p* < 0.05). ***p ≤ 0.001. n.s. = not statistically significant (*p* > 0.05). More extensive results in Web appendix Table A5

As Table 2 shows, trends in smoking have also differently impacted mortality among the low and the high educated. Both smoking and material deprivation are strongly associated with mortality, but only the gap in smoking has widened over time, implying that trends in smoking have probably contributed to widening inequalities in mortality.

**Fig. 4:**
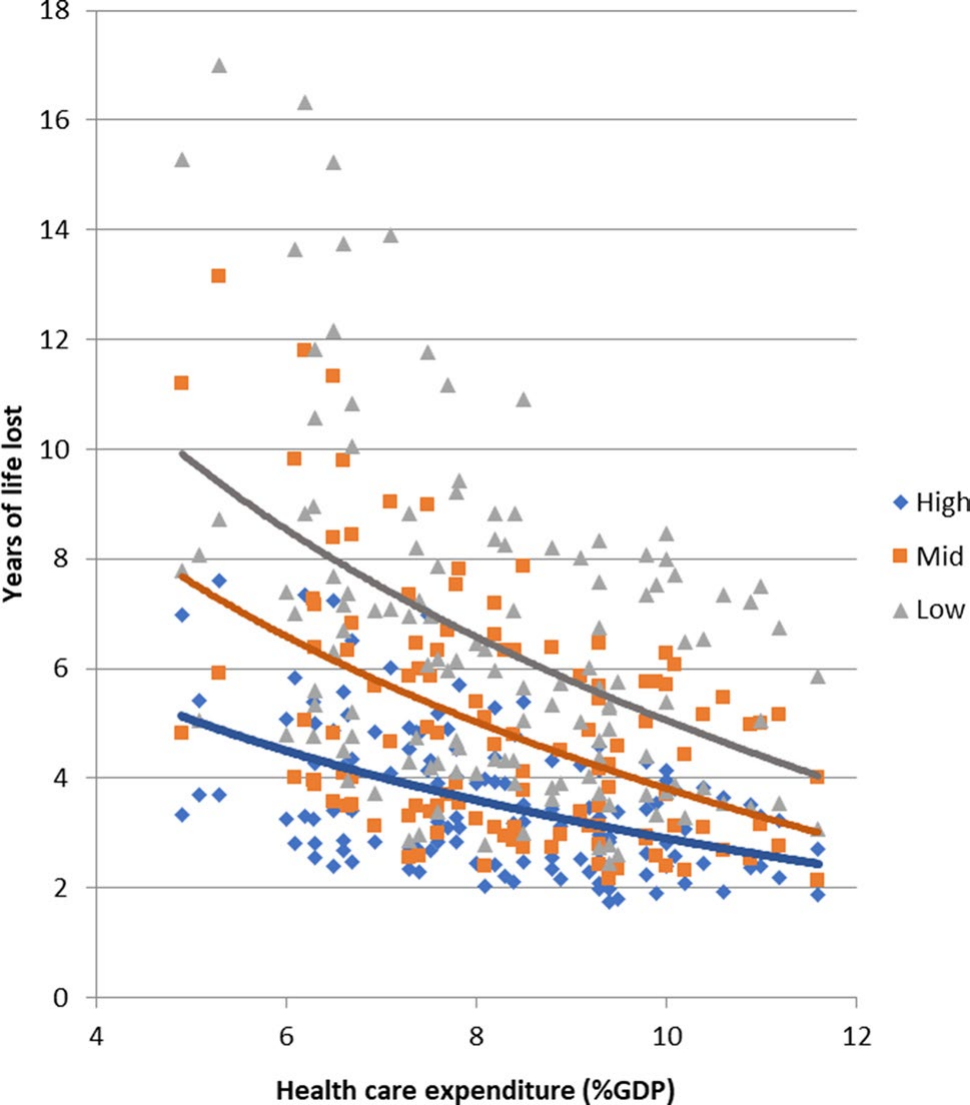
Association between health care expenditure and years of life lost. Notes: Each dot represents one observation (years of life lost by country, period, gender, level of education). Similar results for other mortality measures (mortality rates, life expectancy)

These results show that the narrowing of absolute inequalities in mortality that has occurred in at least some countries, particularly among men, has occurred despite several unfavourable trends, including rising income inequality, transitions towards democracy, and widening inequalities in smoking. The only factor that may have contributed to a narrowing of inequalities in mortality has been rising health care expenditure. This is illustrated in Fig. 4, with years of life lost as the measure of mortality.

At each level of health care expenditure the low educated lose more years than the high educated, but the gap is considerably smaller at higher levels of health expenditure. This is not only seen in a simple correlation diagram as in Fig. 4, but also in country- and period-fixed effects analyses (Table 2). Web Web appendix Table A6 shows that this effect of health care expenditure is seen for many causes of death, but particularly for mortality from amenable causes and cardiovascular diseases.

## Discussion

### Strengths and limitations

This study provides a robust picture of trends in mortality inequalities in a large number of European countries and over a long period of time. Combining different measures of inequalities made us less dependent on the arbitrary choice of a more limited set of measures as used in previous studies. It has recently been argued that when studying trends in health inequalities measures of ‘attainment’ should receive equal attention as measures of ‘shortfall’, which are more commonly used and tend to show widening inequalities [13]. Because ‘attainments’ and ‘shortfalls’ of mortality are more readily conceptualized in terms of years of life lived and lost, we have studied these together with mortality rates. The relationship between life expectancy and age-standardized mortality rates is mathematically complex, and includes a difference in the weight given to different age-groups [17, 18].

Our study shows that trends in ratios of years of life lived are much more favourable than trends in ratios of years lost (Table 1). This is unsurprising in view of the fact that the number of years lived between ages 35 and 80 is much larger than the number of years lost, but our results nevertheless illustrate that whether inequalities in mortality narrow or widen over time is dependent on the measure used. There is no consensus among researchers or policy-makers on what measure of health inequalities to use, and apart from technical considerations value judgements are inevitable when choosing between measures [19]. We have therefore based our rank order of countries on a range of different measures, helped by the fact that this ranking is not too different between measures (Web appendix table A4).

Another major strength of our study is that we have been able to study possible determinants of the magnitude of inequalities in mortality. Studies of determinants of the magnitude of health inequalities at the country-level are almost non-existent, and the only previous study we are aware of studied one point in time only [11]. As our current study exploits time-series data using country- and period-fixed effects, it controls for a wide range of potential confounders. However, because our analyses are ecological in nature, the risk of ecological fallacy prevents us from making causal inferences, particularly about relationships between determinants and mortality at the individual level [20].

Our study has several limitations. For Spain and Italy, only urban and relatively prosperous populations could be included. However, recent national-level studies from Spain and Italy found similarly small inequalities in mortality in the whole country as in these regional populations [21, 22]. Data from Spain, Hungary and Poland were collected in a cross-sectional unlinked design, whereas data from all other countries were collected in a longitudinal design. However, as our main concern is with changes in mortality over time, and as the study design has remained identical, this is unlikely to have biased our findings.

We used level of education as our measure of socioeconomic position, and this does not cover all dimensions of social inequality. However, recent studies of European countries for which more indicators are available, have shown that trends of mortality inequalities by occupational class are largely similar to those by educational level [23]. Our study covers adult ages between 35 and 80 years, implying that the many deaths occurring at higher ages are not included, and neither are deaths occurring at younger ages for which trends and contributing causes may partly be different.

### Interpretation

The challenge facing countries that aim to reduce inequalities in mortality in a context of rapid mortality decline, is to make sure that the mortality decline in lower socioeconomic groups exceeds that of higher socioeconomic groups. The general rise of relative inequalities in mortality shows that almost no European country has achieved this for relative (i.e., percentage) mortality declines. On the other hand, many European countries have achieved this for absolute mortality declines, particularly among men, as shown by a decline of absolute inequalities in mortality. It can also be mathematically demonstrated that a narrowing of absolute inequalities occurs under a much wider range of conditions, in terms of combinations of changes to mortality in lower and higher socioeconomic groups, than a narrowing of relative inequalities in mortality [24].

It is encouraging to see that some European countries have achieved absolute reductions in mortality among the low educated strongly exceeding those among the high educated. This applies, first of all, to some Eastern European countries, particularly Hungary and Estonia. Although the period of mortality decline covered by our analysis is still rather short, the achievements of both countries are spectacular. The recent declines in average mortality in Eastern Europe have been attributed to a combination of long-term declines in smoking, improvements in health care, dietary changes, road traffic safety measures and alcohol control policies [25]. Apparently, Hungary and Estonia have managed to bring the benefits of these mortality-lowering policies also to the low educated.

However, some Western European countries have also done very well, particularly Austria, England and Wales, Italy (Turin), Spain (Barcelona) and Switzerland. Our cause-specific mortality results suggest that the reductions in absolute inequalities in mortality have been achieved as a by-product of population-wide improvements in prevention and treatment, particularly for ischemic heart disease, smoking-related causes, and causes amenable to medical intervention. In the case of ischemic heart disease, these trends must be due to more favourable changes in either ‘proximate’ determinants of ischemic heart disease, such as health-related behaviours (e.g., smoking, diet, exercise) or health care effectiveness (e.g., hypertension detection and treatment, thrombolytic therapy), or both, among the low than the high educated. A recent narrowing of absolute inequalities in cardiovascular disease mortality has previously been reported for both England and Scotland, and has been attributed to an even distribution of treatment benefits rather than to risk factor changes [26].

The worst performers, both among men and among women, are three Nordic countries: Finland, Norway and Denmark. We have demonstrated previously in cross-sectional analyses that, unlike what one would expect on the basis of their universal and generous welfare states, these countries do not have smaller health inequalities than other European countries [27]. This new analysis shows that over the past decades, these countries have also experienced relatively unfavourable trends in inequalities in mortality. This ‘Nordic’ paradox has a complex explanation, including further advanced changes in social stratification and social mobility than elsewhere in Europe; further advanced changes in smoking, fertility behaviour and other proximal determinants of population health; and stubborn persistence of inequalities in social disadvantage [28].

Our study of determinants of mortality inequalities showed that trends over the past 25 years in several determinants may have contributed to a widening of inequalities in mortality. Income inequality has gone up [29], and this has been associated with rising mortality, but more so among the low than the high educated, suggesting an inequalities-widening effect. Levels of democracy have gone up in Eastern Europe in the 1990s, and the accompanying political and economic turbulence has been associated with a temporary rise in mortality, but more so among the low than the high educated, as a result of their greater vulnerability to increased economic stress and disruption of social and health care services [30]. Smoking has mostly gone down, but more so among the high than among the low educated [31], again implying an inequalities-widening effect.

Only rising health care expenditure was associated with smaller absolute inequalities in mortality. In a more detailed analysis reported elsewhere we have shown that this effect applies particularly to causes amenable to medical care, and not to non-amenable causes [32]. These results were obtained with regression models controlling for national income and country- and period-fixed effects, and are therefore robust against various sources of confounding.

This suggests that in many European countries health care expansion, in response to greater demand and enlarged opportunities for medical intervention, has not only contributed to average mortality decline as shown elsewhere [33], but may also have had a dampening effect on inequalities in mortality. Even though it would be better to tackle the root causes of health inequalities [34], this is good news, and shows that the role of health care in reducing, or simply containing, health inequalities should not be ignored.

## Electronic supplementary material

Below is the link to the electronic supplementary material.

Supplementary material 1 (DOCX 107 kb)

## References

[1] Mackenbach JP, Stirbu I, Roskam AJ (2008). Socioeconomic inequalities in health in 22 European countries. N Engl J Med.

[2] Toch-Marquardt M, Menvielle G, Eikemo TA (2014). Occupational class inequalities in all-cause and cause-specific mortality among middle-aged men in 14 European populations during the early 2000s. PLoS ONE.

[3] Mortensen LH, Rehnberg J, Dahl E (2016). Shape of the association between income and mortality: a cohort study of Denmark, Finland, Norway and Sweden in 1995 and 2003. BMJ Open.

[4] Mackenbach JP (2012). The persistence of health inequalities in modern welfare states: the explanation of a paradox. Soc Sci Med.

[5] Mackenbach JP, Kulhánová I, Artnik B (2016). Changes in mortality inequalities over two decades: register based study of European countries. Br Med J.

[6] Galobardes B, Lynch J, Smith GD (2007). Measuring socioeconomic position in health research. Br Med Bull.

[7] Mackenbach JP, Looman CWN (2013). Life expectancy and national income in Europe, 1900-2008: an update of Preston’s analysis. Int J Epidemiol.

[8] Pickett KE, Wilkinson RG (2015). Income inequality and health: a causal review. Soc Sci Med.

[9] Mackenbach JP, Hu Y, Looman CWN (2013). Democratization and life expectancy in Europe, 1960–2008. Soc Sci Med.

[10] van Baal P, Obulqasim P, Brouwer W, Nusselder W, Mackenbach J (2013). The influence of health care spending on life expectancy.

[11] Mackenbach JP, Bopp M, Deboosere P (2017). Determinants of the magnitude of socioeconomic inequalities in mortality: a study of 17 European countries. Health Place.

[12] Ahmad OB, Boschi-Pinto C, Lopez AD, Murray CJL, Lozano R, Inoue M (2001). Age standardization of rates: a new WHO standard.

[13] Kjellsson G, Gerdtham U-G, Petrie D (2015). Lies, damned lies, and health inequality measurements: understanding the value judgments. Epidemiology.

[14] Mackenbach JP, Kunst AE (1997). Measuring the magnitude of socio-economic inequalities in health: an overview of available measures illustrated with two examples from Europe. Soc Sci Med.

[15] Moreno-Betancur M, Latouche A, Menvielle G, Kunst AE, Rey G (2015). Relative index of inequality and slope index of inequality: a structured regression framework for estimation. Epidemiology.

[16] Latouche A, Andersen PK, Rey G, Moreno-Betancur M (2019). A note on the measurement of socioeconomic inequalities in life years lost by cause of death. Epidemiology.

[17] Keyfitz N, Caswell H (2005). Applied mathematical demography.

[18] Vaupel JW (1986). How change in age-specific mortality affects life expectancy. Popul Stud.

[19] Harper S, King NB, Meersman SC, Reichman ME, Breen N, Lynch J (2010). Implicit value judgments in the measurement of health inequalities. Milbank Q.

[20] Piantadosi S, Byar DP, Green SB (1988). The ecological fallacy. Am J Epidemiol.

[21] Marinacci C, Grippo F, Pappagallo M (2013). Social inequalities in total and cause-specific mortality of a sample of the Italian population, from 1999 to 2007. Eur J Public Health.

[22] Regidor E, Kunst AE, Rodriguez-Artalejo F, Mackenbach JP (2012). Small socio-economic differences in mortality in Spanish older people. Eur J Public Health.

[23] de Gelder R, Menvielle G, Costa G (2017). Long-term trends in socioeconomic inequalities in mortality in 6 European countries. Int J Public Health.

[24] Mackenbach JP, Martikainen P, Menvielle G, de Gelder R (2016). The arithmetic of reducing relative and absolute inequalities in health: a theoretical analysis illustrated with European mortality data. J Epidemiol Community Health.

[25] Mackenbach JP, Karanikolos M, Lopez Bernal J, Mckee M (2015). Why did life expectancy in Central and Eastern Europe suddenly improve in the 1990s? An analysis by cause of death. Scand J Public Health.

[26] Bajekal M, Scholes S, Love H (2012). Analysing recent socioeconomic trends in coronary heart disease mortality in England, 2000–2007: a population modelling study. PLoS Med.

[27] Mackenbach JP, Kunst AE, Cavelaars AE, Groenhof F, Geurts JJ (1997). Socioeconomic inequalities in morbidity and mortality in western Europe. Lancet.

[28] Mackenbach JP (2017). Nordic paradox, southern miracle, eastern disaster: persistence of inequalities in mortality in Europe. Eur J Public Health.

[29] OECD (2011). Divided we stand: why inequality keeps rising.

[30] Leinsalu M, Stirbu I, Vagero D (2009). Educational inequalities in mortality in four Eastern European countries: divergence in trends during the post-communist transition from 1990 to 2000. Int J Epidemiol.

[31] Giskes K, Kunst AE, Benach J (2005). Trends in smoking behaviour between 1985 and 2000 in nine European countries by education. J Epidemiol Commun Health.

[32] Mackenbach JP, Hu Y, Artnik B (2017). Trends In inequalities in mortality amenable to health care in 17 European countries. Health Aff.

[33] Heijink R, Koolman X, Westert GP (2013). Spending more money, saving more lives? The relationship between avoidable mortality and healthcare spending in 14 countries. Eur J Health Econ.

[34] Commission on Social Determinants of Health (2008). Closing the gap in a generation. Health equity through the social determinants of health.

