# Supplementary material for: Progress against inequalities in mortality: register-based study of 15 European countries between 1990 and 2015
Source: Eur J Epidemiol. 2019 Nov 15;34(12):1131–42. doi: 10.1007/s10654-019-00580-9 (PMC7010632; doi:10.1007/s10654-019-00580-9)
Supplement: Supplementary file 1 — Supplementary material 1 (DOCX 107 kb) [file 10654_2019_580_MOESM1_ESM.docx]

# Web appendix

**Table A1. Overview of mortality data sources**

**Table A2. ICD-codes for the causes of death included in the analysis**

**Table A3. Overview of mortality rates and life expectancy values**

**Table A4. Rank order of countries on eight measures of relative improvement of inequalities in mortality/life expectancy among the low as compared to the high educated**

1. **Men**

1. **Women**

Notes: For each inequality measure, rank orders of countries (with 1 representing the country with most narrowing or least widening of inequalities) were based on ‘relative’ or percentage changes between the beginning and end of the observation period. Because of their shorter observation periods, rank orders of countries in Eastern Europe were determined separately. In green: best performers; in orange: worst performers.

**Table A5. Results of regression analyses for determinants of inequalities in total mortality**

**Table A6. Results of regression analyses for the effect of health care expenditure on inequalities in cause-specific mortality**
